# Evaluation of Anxiety and Depression in a Community Sample of Transgender Youth

**DOI:** 10.1001/jamanetworkopen.2021.4739

**Published:** 2021-04-07

**Authors:** Dominic J. Gibson, Jessica J. Glazier, Kristina R. Olson

**Affiliations:** 1 University of Washington, Seattle; 2 Princeton University, Princeton, New Jersey

## Abstract

This cross-sectional study of families with transgender children compares responses on anxiety and depression questionnaires from transgender youth, cisgender siblings of transgender children, and cisgender age-matched controls, as well as the parents of all 3 groups.

## Introduction

Most studies have found that youth who do not conform to gender norms for their assigned sex have higher rates of depression and anxiety than their cisgender peers.^1,2^ However, more recent research featuring smaller cohorts (ie, ranging from 31 to 73 participants) of socially transitioned transgender youth—youth who identify and live as a gender different from their sex assignment at birth—show normative or only slightly elevated rates of depression and anxiety.^3,4,5^ We recruited a new, larger sample of socially transitioned transgender youth, their siblings, and age- and gender-matched control participants to test whether transgender youth experience significantly higher levels of anxiety and depression than their cisgender peers.

## Methods

This cross-sectional study includes responses from 3 groups of youth between ages 8 and 14 years in a large community-recruited sample (following previous recruitment strategies^3,5^): transgender youth (148 participants), cisgender siblings of transgender children (88 participants), and cisgender age-matched controls (139 participants). We obtained written consent from parents and verbal and/or written assent from children. This study was approved by the University of Washington institutional review board. This study followed the Strengthening the Reporting of Observational Studies in Epidemiology (STROBE) reporting guideline.

We measured depression and anxiety using the pediatric short form (completed by youth) and proxy form (completed by parents) of the National Institutes of Health’s Patient Reported Outcomes Measurement Information System (PROMIS) scale, which measures depression and anxiety on a 100-point scale where 50 is the population mean and 40 to 60 is the reference range (see eAppendix in the Supplement). Participants must have completed 1 or both self-reported mental health measures between November 2014 and March 2020, and none of the youth in this article ever reported on their own mental health in any other study. Fifty-two parents reported on their children in a previously published report (50 at a different time point and 2 at the same time point as in this report).^5^ Data were analyzed from July to November 2020 and used R version 3.6 (R Project for Statistical Computing). *P* < .05 was considered significant and all tests were 2-sided.

## Results

A total of 375 participants (124 girls [33.1%], 251 boys [66.9%]; 267 [71.2%] White participants, 15 [4.0%] Asian participants, 7 [1.9%] Black participants, 7 [1.9%] Hispanic participants, and 69 [18.4%] participants who identified as multiracial/other) between the ages of 8 and 14 years (mean [SD] age, 10.54 [1.05] years) and their parents were included (Table 1). Means, SDs, frequencies, and percentages of children who scored within the clinical range on each measure are reported in Table 2. One-way analyses of variance for each of the measures revealed no significant group differences in self-reported depressive symptoms, self-reported anxiety symptoms, or parent-reported depressive symptoms. Parent-reported anxiety differed significantly by group. Post hoc Tukey tests showed that parents reported higher rates of anxiety in transgender youth than in control participants (mean [SD] PROMIS score: 52.62 [9.41] vs 49.94 [8.84]; *P* = .04, d = 0.29) but not siblings (50.23 [9.32] vs 49.94 [8.84]; *P* = .13, d = 0.25). Siblings did not differ from control participants (52.41 [8.82] vs 50.53 [8.25]; *P* = .97, d = 0.03).

**Table 1.** Demographic Characteristics by Group

Characteristic	Children, No. (%)		
	Transgender (n = 148)	Sibling (n = 88)	Control (n = 139)
Gender identity			
Boys	53 (36)	47 (53)	48 (35)
Girls	95 (64)	41 (47)	91 (65)
Race or ethnicity			
Asian	3 (2)	3 (3)	9 (6)
Black	3 (2)	1 (1)	3 (2)
Hispanic/Latino	4 (3)	5 (6)	0
Native American/Alaskan Native	1 (1)	1 (1)	1 (1)
Multiracial/other^a^	18 (12)	15 (17)	33 (24)
White, non-Hispanic	114 (77)	61 (69)	92 (66)
Missing	5 (3)	2 (2)	1 (1)
Age, mean (SD), y	10.1 (1.0)	10.2 (1.2)	10.1 (1.0)
Income, $			
<25 000	5 (3)	3 (3)	2 (1)
25 001-50 000	13 (9)	6 (7)	5 (4)
50 001-75 000	26 (18)	13 (15)	8 (6)
75 001-125 000	33 (22)	29 (33)	38 (27)
>125 000	71 (48)	37 (42)	84 (60)
Missing	NA	NA	2 (1)

Abbreviation: NA, not applicable.

^a^ Two participants only listed other for race/ethnicity; the rest listed multiple races.

**Table 2.** Mean Score Estimates and Comparisons by Group for Each of the 4 Measures

Group	PROMIS score estimates		Group comparison		
	Mean (SD) score^**a**^	Children scoring in clinical range, No. (%)^**b**^	*F* test	*P* value	η^2^
**Child-reported depression**					
Transgender	46.38 (9.13)	5 (3.4)	*F*_2371_ = 1.03	.36	0.01
Sibling	48.01 (9.05)	4 (4.6)			
Control	46.46 (8.99)	3 (2.2)			
**Child-reported anxiety**					
Transgender	52.21 (8.92)	17 (11.5)	*F*_2372_ = 1.81	.17	0.01
Sibling	52.41 (8.82)	12 (13.6)			
Control	50.53 (8.25)	9 (6.5)			
**Parent-reported depression**^c^					
Transgender	51.41 (8.06)	12 (8.1)	*F*_2372_ = 1.45	.24	0.01
Sibling	51.1 (8.52)	8 (9.1)			
Control	49.86 (7.65)	6 (4.3)			
**Parent-reported anxiety**^c^					
Transgender	52.62 (9.41)	20 (13.5)	*F*_2372_ = 3.55	.03	0.02
Sibling	50.23 (9.32)	6 (6.8)			
Control	49.94 (8.84)	11 (7.9)			

^a^ These measures are normed such that the mean (SD) score of 50 (10) is the national average. Lower scores indicate lower depression or anxiety.

^b^ Clinical range defined as a score of ≥63, representing approximately 10% of a representative sample of youth in the age range on these measures.

^c^ When 2 parents provided responses for a given child, their scores were averaged.

## Discussion

Our results were consistent with recent smaller studies of socially transitioned transgender youth and contrast with the more severe mental health symptoms found in earlier studies of youth referred to clinics for gender-related concerns (as well as transgender teens and adults^6^), although these samples differ in many ways (eg, date and location of data collection, clinic-recruited vs community samples, the present children had socially transitioned) that make direct comparisons difficult.

This study did have limitations. Although the present sample was larger than previous studies of transgender youth, it likely overrepresented families with higher levels of parental education, higher socioeconomic status, that are White, and other factors. Whether these biases reflect who is socially transitioning at the time of the study is unknown.

Nonetheless, these results demonstrate that many socially transitioned transgender youth experience levels of anxiety and depression in the normative range and equal to or only slightly higher than siblings and cisgender peers. Whether their generally strong mental health is because of their early social transition, the high levels of support they receive, or other factors is as yet unknown. The current findings do not negate the experiences of the many transgender people who face high rates of mental health challenges^6^ but do provide further evidence that being transgender is not synonymous with these challenges.

## Notes

Supplement. **eAppendix.** Participants and Procedures
