# Error in Results Section

**DOI:** 10.1001/jamanetworkopen.2021.13597

**Published:** 2021-05-11

In the Research Letter titled “Evaluation of Anxiety and Depression in a Community Sample of Transgender Youth,”^1^ published April 7, 2021, there was an error in the demographic information provided in the first sentence of the Results section. The gender distribution of the study participants should have been reported as 227 girls (60.5%) and 148 boys (39.5%). The article has been corrected.
