# Supplementary material for: Evaluation of Anxiety and Depression in a Community Sample of Transgender Youth
Source: JAMA Netw Open. 2021 Apr 7;4(4):e214739. doi: 10.1001/jamanetworkopen.2021.4739 (PMC8027909; doi:10.1001/jamanetworkopen.2021.4739)
Supplement: Supplement. — eAppendix. Participants and Procedures [file jamanetwopen-e214739-s001.pdf]

Supplemental Online Content

Gibson DJ, Glazier JJ, Olson KR. Evaluation of anxiety and depression in a community sample of transgender youth. *JAMA Netw Open*. 2021;4(4):e214739. doi:10.1001/jamanetworkopen.2021.4739

eAppendix. Participants and Procedures

This supplemental material has been provided by the authors to give readers additional information about their work.

© 2021 Gibson DJ et al. *JAMA Network Open.*

eAppendix. Participants and Procedures

Participants

Participants were recruited as part of two larger research studies. Most participants (n=330) were part of an ongoing longitudinal study of transgender youth, their siblings, and control participants, called the TransYouth Project. During the period of data collection for the current paper, but after the TYP cohort had completed recruitment, 45 additional families with children in the same age range, recruited through the same means, completed the same measures and therefore are also included in the present analyses.

Socially-transitioned transgender children and their siblings were recruited through word of mouth, national and local support groups, summer camps, and forums for families of transgender and gender-nonconforming youth. The research team traveled around the U.S. and parts of Canada to meet with transgender participants. To be included as a transgender participant in the current study, children needed to use the pronouns "opposite" their assigned sex at birth in all contexts (e.g., at school, at home, in new environments) which is often called a complete or a full "social transition." Siblings of transgender children were recruited and participated at the same time as their transgender siblings; however not all transgender participants had siblings or had siblings in the appropriate age range and therefore the number of sibling participants is smaller than the number of transgender participants.

For each transgender participant, an age- and gender-matched control was recruited through a university database of families who expressed interest in participating in child development research. Controls all came from the Pacific Northwestern United States. In addition to these child participants, the parents of these participants also participated in the current study. While one control was typically recruited for each transgender participant, there

© 2021 Gibson DJ et al. *JAMA Network Open.*

are slightly fewer controls than transgender participants in the present study because 3 recruited controls did not complete the tasks described in this study, 4 controls have not yet been tested (and due to the COVID-19 pandemic it is unclear when they will be able to participate), and due to experimenter error, 1 transgender participant's corresponding control participant was never tested.

All participants completed a much larger battery of measures, most of which had to do with other aspects of development (e.g., preferences for gendered clothing, views about gender); the measures reported in this manuscript were the only mental health measures. Participants were only included in the present report if they completed at least one of the self-reported mental health measures described below. If children completed these mental health measures at multiple visits (as part of the larger longitudinal study), only data from the earliest visit were included. Parent-reported data always came from the same visit as the child-reported data. The present sample includes data from the first visit of 177 (47%) participants, the second visit of 165 (44%) participants, and the third visit of 33 (9%) participants. When data came from later visits this was because no mental health data had been assessed in children before that visit (e.g., because children were too young to complete the measures, because children had quit the study before getting to the mental health measures, or because the measures had not been added to the battery at the time of their first or second visit--assessing mental health was not the primary purpose of the original study).

To be included in the current report, child participants must have completed one or both of the self-report mental health measures between November 2014 and March 2020 and could not have been reported in the child's mental health data in Durwood et al (2017). The exact data from 2 children's parents reported in the current paper are also reported in Olson et al (2016); 50

other children's parents reported on the same children but at a different time point in Olson et al, 2016; the parent-reported depression and anxiety of the remaining 323 children in this paper have never been reported in any other paper. None of the children in this paper have ever reported on their own mental health in any other paper.

The present sample included 148 transgender children, 139 matched controls, and 88 siblings between the ages of 8 and 14 years and their parents (children below age 8 who participated in the broader survey did not complete these measures). One participant completed the self-reported anxiety scale but not the self-reported depression scale and was thus left out of analyses of the self-reported depression scale. Demographics for each group are presented in Table 1 in the main document.

Procedure

Children and their parents completed the measures either in separate rooms or at a distance from one another in the same room (in their homes, our laboratory, or other public spaces such as at conferences for families with transgender children) so as not to influence each other's responses. For participants younger than 12 years old, an experimenter read each question out loud and children gave either verbal responses or pointed to their response on a scale. Parents and older children completed written versions of the measures with experimenters available nearby to answer questions.

Measures

We measured children's self-reported depression (α = 0.86) and anxiety (α = 0.85) symptoms using the pediatric short form of the National Institutes of Health's Patient Reported

© 2021 Gibson DJ et al. *JAMA Network Open.*

Outcomes Measurement Information System (PROMIS) scales. Parents completed the proxy versions of the PROMIS depression (parent 1, $\alpha = 0.89$; parent 2, $\alpha = 0.9$) and anxiety (parent 1, $\alpha = 0.93$; parent 2, $\alpha = 0.93$) scales. On average these measures were taken 2.8 years (*SD*=1.6) after transgender participants had transitioned.

Each scale is made up of items such as "I felt worried" or "I felt unhappy" and participants were asked to indicate how often they (or their children) felt that way in the past 7 days by selecting one of five options: "never," "almost-never," "sometimes," "often," "very often". Responses were converted to a Likert-type scale, summed, and converted to standardized T scores, which are normed such that a score of 50 is the national average for all youth with a standard deviation of 10 points. Scores greater than or equal to 63 are considered clinically significant as they are within approximately the top 10% of all youth.

Of the 375 youth in the study, 140 had two parents complete the parent proxy measures and the remaining 235 had only one parent complete the measures. In line with previous work, when two parents completed the measures, their scores were averaged. Parents' responses tended to be related (depression: $r=.41$, $p<.001$, $n=140$; anxiety: $r=.45$, $p<.001$, $n=140$). When we run the analyses with only the one parent's report per child, the results remain largely unchanged with one exceptions: the difference in parent-reported anxiety for transgender children and siblings becomes significant ($p=.035$). This difference is consistent with our conclusions that the transgender participants in our sample experience levels of anxiety and depression in the normative range and equal to or only slightly higher than their siblings and cisgender peers.

© 2021 Gibson DJ et al. *JAMA Network Open.*
